# Thyroid metastasis from chondrosarcoma

**DOI:** 10.1097/MD.0000000000018043

**Published:** 2019-11-22

**Authors:** Zhi-Hong Wu, Jin-Yao Dai, Jia-Ni Shi, Mei-Yu Fang, Jun Cao

**Affiliations:** a Oncology Department, Yiwu Central Hospital, Jinhua; b Second Clinical Medical College, Zhejiang Chinese Medical University; c Third Clinical Medical College, Zhejiang Chinese Medical University; d Department of Comprehensive Medical Oncology, Key Laboratory of Head and Neck Cancer Translational Research of Zhejiang Province, Cancer Hospital of University of Chinese Academy of Sciences, Zhejiang Cancer Hospital, Hangzhou, Zhejiang, China.

**Keywords:** chondrosarcoma, metastasis, thyroid gland, treatment

## Abstract

For chondrosarcoma, metastasis to the thyroid gland is extremely rare. The diagnosis and treatment of thyroid metastasis from chondrosarcoma are discussed here.

We found a case of thyroid malignancy occurring after treatment of chondrosarcoma. We reviewed patient characteristics, histological presentations on initial chondrosarcoma and thyroid metastasis, treatments, times of recurrence and death. In addition, we searched Embase, PubMed, and ISI Web of Science databases (1996–2018) for articles published in the English language using the key words “chondrosarcoma” and “thyroid” and we reviewed almost all the reports about thyroid metastasis from chondrosarcoma.

Only 5 cases of chondrosarcoma metastases in the thyroid gland have been reported in the literature. We found that most patients are adults, with compression signs or pain, most of whom have poor prognoses. The main examinations are ultrasound, CT and fine needle aspiration biopsy, and primary treatment is surgery.

These rare cases of chondrosarcoma presenting as a metastasis in the thyroid gland highlight the importance of close communication between radiologists, histopathologists, and clinicians to ensure that such exceptional cases are not missed.

## 1 Introduction

Clinically, metastases to the thyroid gland are rarely observed. The most frequent primary sites of thyroid metastases are reported to be kidney, followed by breast and lung.^[1–2]^ Chondrosarcoma is a very complicated disease that does not have a clear genetic background or pathogenesis. The metastasis of chondrosarcoma is mainly hematogenous. The vast majority of metastases occur in the lungs,^[3]^ with metastasis to the thyroid gland being extremely rare. Whereas the association between chondrosarcoma and thyroid carcinoma has not been recognized at present, vigilant surveillance and close follow-up should be emphasized for all patients. To our knowledge, only 5 cases of thyroid metastases from chondrosarcomas have been reported. Here, we present a case in which the patient developed metastasis to the thyroid gland 4 years later after chondrosarcoma therapy.

## 2 Patients and methods

**Figure 1:**
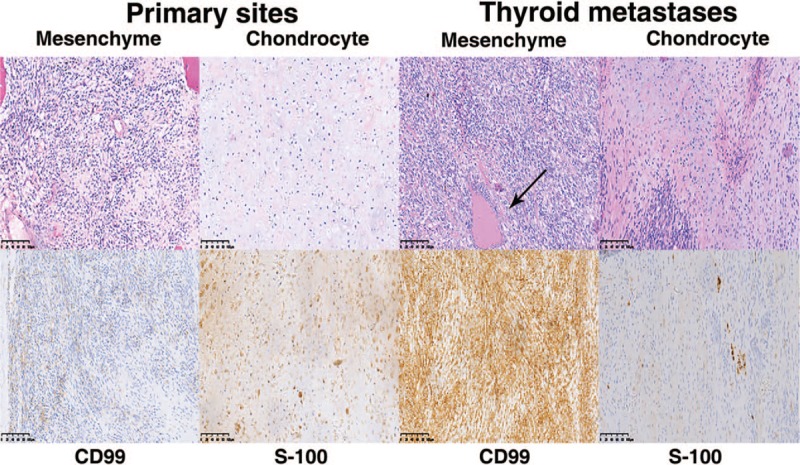
Hematoxylin and eosin staining and immunohistochemistry of thyroid metastases originating from chondrosarcoma. The distributions of chondrocytes were island-like and relatively mature. The cells varied in size and shape, with calcification or ossification and were positive for S-100. Mesenchymal cells showed as sheets, oval and short fusiform. Most were undifferentiated. And the cells were positive for cluster of differentiation 99 (CD99) and neuron-specific enolase. The arrow pointed remnants thyroid follicle.

A 51-year old woman came to the local hospital in March 2009 because of a mass in her left thigh, about 2.5 cm in diameter. She underwent resection of mass in the left thigh, and the pathology revealed the diagnosis of spindle cell tumors, first considering mesenchymal chondrosarcoma. Then she came to our hospital for further diagnosis and the mesenchymal chondrosarcoma was confirmed. She was treated with the extensive resection of the left thigh on tumor bed, with a consistent clear border. The recovery after surgery was functional, and the patient was reexamined regularly. In 2010, she presented some bean-size, painless masses in her left thigh. Then the left mass resection was performed. Histologically, the left thigh lesion showed local recurrences. Consequently, she received extensive resection and radiotherapy (first stage: DT 40 Gy/20 F, second stage: DT 20 Gy/10 F). In 2012, on a routine checkup, ultrasound examination was remarkable for many nodules in her thyroid gland. She denied local or specific symptoms of thyroid. Fine needle aspiration biopsy (FNAB) guided by ultrasonography of the left thyroid nodules revealed that a few follicular epithelium, colloid and lymphocytes were scattering distribution. In November 2013, she underwent right subtotal thyroidectomy and left lobectomy. The final pathology indicated the diagnosis of mesenchymal chondrosarcoma metastasis in her thyroid gland. Thyroid lesion was composed of chondrocytes and mesenchymal cells. Chondrocytes were island-like, relatively mature and varied in size and shape, with calcification or ossification. Mesenchymal cells most were undifferentiated, showing as sheets, oval and short fusiform (Fig. 1). Immunohistochemical staining showed that chondrocytes were strongly positive for S-100 and undifferentiated mesenchymal cells were strongly positive for CD99 and NSE. Meanwhile, ultrasound and computed tomography (CT) showed that hypoechoic left liver inner lobe, fatty liver, small cysts in the right kidney, as well as nodules in the left lung. One year later, radiologic evaluation, including X-ray, CT and magnetic resonance imaging (MRI), revealed the presence of nodules and masses in her lungs, spleen, pancreas and peritoneal cavity, most consistent with metastatic disease from her primary chondrosarcoma. The patient received no further therapy due to the tumor's extensive metastases and died in the March of 2015.

By observing and collecting case reports, we summarized and analyzed the characteristics of the case reports. We also searched Embase, PubMed, and ISI Web of Science databases for articles published in the English language using the key words “chondrosarcoma” and “thyroid”, and we reviewed almost all reports describing thyroid metastasis from chondrosarcoma. We reviewed medical records and reported the following data: the demographic data, presenting symptoms, treatment of primary and secondary carcinoma, and survival time (Table 1). Based on the literature review, we correspondingly analyzed and extrapolated, exploring the new findings and applying the value in clinical practice. This study was approved by the Ethics Committee of Zhejiang Cancer Hospital in Hangzhou, China, and the patient signed informed consent.

**Table 1:**
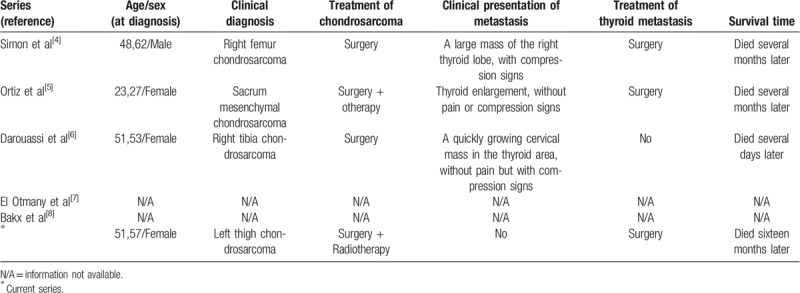
Summary of reported cases of thyroid metastasis from chondrosarcoma.

## 3 Results

Table 1 summarizes the clinical circumstances of the 5 cases previously published and our report of thyroid metastasis from chondrosarcoma.^[4–8]^ The ages of the patients at presentation are variable, ranging from 27 to 62 years with an average age of 47.3 years. Most patients present symptoms, such as dysphagia and dyspnea. The main examinations are ultrasound, CT, and FNAB, and every patient underwent at least one of these examinations. Furthermore, surgeries are received by most patients. Nevertheless, the prognosis is generally poor, and the survival time of 5 patients at presentation is variable, ranging from several days to several months.

## 4 Discussion

The incidence of metastasis to the thyroid gland has been thought to be as low as 0.05%.^[9]^ The thyroid gland can be involved by direct invasion from adjacent structures, hematogenous spread and lymphatic spread.^[10]^ The most common primary sites are kidney carcinoma, followed by breast carcinoma, lung carcinoma, melanoma and colon and laryngeal carcinoma.^[1–2]^ Thyroid metastases may appear many years or decades after primary malignancies, making the diagnosis of metastases ignored. Furthermore, secondary thyroid malignancy manifests itself as a primary thyroid tumor, and may clinically mislead clinicians, pathologists and radiologists. In 1931, Willis proposed hypotheses^[11]^: thyroid glands with fast blood flow, the high oxygen saturation and iodine content discourage the growth of malignant cells. That is the reason why the frequency of intra thyroid metastases is relatively low. Although thyroid has this self-protection, the richly vascularized thyroid is most likely to be transferred by micro tumor cells in blood.^[6]^ Chondrosarcomas are a heterogeneous group of malignant cartilaginous neoplasms, which account for 20% to 25% of all primary bone malignancies.^[12]^ The metastasis of chondrosarcoma is predominantly hematogenous. Distant metastases to the lung are the most common, followed by skin and soft tissue, while metastasis to the thyroid gland is extremely infrequent.^[3]^

At present, there have been 5 cases of chondrosarcoma metastases to the thyroid gland reported (Table 1). Francois Simon^[4]^ reported a case of right femur chondrosarcoma. Fourteen years later, this patient presented a large mass in his right thyroid lobe and received surgery. Ortiz^[5]^ reported metastatic mesenchymal chondrosarcoma, including thyroid and bone metastasis. Darouassi^[6]^ reported a case of a 51-year-old woman presented chondrosarcoma in right tibia. Thyroid carcinoma developed 2 years after the treatment of chondrosarcoma.

In these reports, thyroid metastases occur 2 to 14 years after the primary chondrosarcoma. Chondrosarcomas often appear in patients aged between 30 and 60 years old, with a preference in males.^[13]^ However, many cases of younger patients have been reported.^[14]^ In this review, the evidence that the youngest age of onset is 23 confirms the conclusion. Chondrosarcomas usually occur in the pelvis, proximal long bones, ribs, scapulae, and vertebras.^[15]^ Meanwhile, most of these patients were treated with surgery. Only 1 patient received chemotherapy after surgery. Surgery can be taken as the prior choice in the treatment of chondrosarcoma. The patient we reported was treated by radiotherapy after surgery, because of local recurrence 1 year later, whose treatment was different from most reported cases. Radiation can be used in the situation where resection is not complete and feasible, aiming at maximal local control.^[16]^ Clinical presentation of metastasis is mainly palpable thyroid node, with dysphagia, dyspnea, etc. Our patient had no obvious symptoms due to early discovery. Besides, surgeries were received by most patients when thyroid metastases happening. Surgery included total thyroid thyroidectomy, lobectomy, tracheostomy and was not effective in prolonging survival time.

All case reports have suggested that patients had poor prognoses with metastases to the thyroid gland. In our case, some possible reasons accounted for it. For one thing, the patient was 51 years old and metabolism function became lower; for another, widely metastases from chondrosarcoma influenced liver, lung, spleen, and pancreas so that disease was rapidly worse. Other reported cases are also associated with multifocal metastases, involved lung metastasis and bone metastasis. Patients with a single metastasis within the thyroid have better survival than those with multiple metastases.^[17]^

Clinical, diagnosis was made by histopathology examination and surgery.^[10,18]^ Thyroid palpable nodules in a patient with a history of malignancy can suggest thyroid metastasis, especially when patient presenting many years after the initial carcinoma. FNAB which has low cost and high negative value is recommended in diagnosing the thyroid metastases. However, it may be difficult to diagnose thyroid metastasis occasionally, such as unclear malignancy history and anaplastic carcinoma. Hence, thyroidectomy specimen and pathology examination are necessary and effective. Furthermore, immunohistochemical markers are useful diagnostic tools in differentiating between primary thyroid malignancy and secondary malignancy. Immunohistochemical markers like CD99 and NSE are suggested in diagnosing known primary chondrosarcoma. Positive staining with anti-thyroglobulin and anti-calcitonin antibodies would favor a primary thyroid tumor.^[10]^

Currently, completely surgical excision is the only treatment proven to be effective for chondrosarcoma. Other therapies include chemotherapy, radiotherapy, and targeted therapy. It is widely believed that chondrosarcomas are resistant to both chemotherapy and radiation, due to their poor vascularity, extracellular matrix and the low percentage of dividing cells.^[19]^ Targeted therapy is a new focus and clinical studies are ongoing. The expression of the antiapoptotic (BCL2), protein kinase C (PKC-α), and platelet-derived growth factor receptor (PDGFR-α) pathways was found, which suggests potential targets for mesenchymal chondrosarcoma therapy.^[20]^ The majority of chondrosarcomas grow slowly and have good prognoses after adequate surgery. However, it has been reported that metastases occur in about 22% to 32% of patients, most of whom have poor prognoses.^[21]^ On histological examination, conventional chondrosarcoma has been recognized that histological grading correlates with prognosis. It is classified into 3 histological grades (Grade I, II, and III). Grade II and III conventional chondrosarcomas have poorer prognoses, which are respectively associated with metastatic rates of 10% and 71%.^[22]^ In our case, the mesenchymal chondrosarcoma, which is a rare malignant tumor and accounts for less than 3% of primary chondrosarcomas, is characterized by relatively common originating from soft tissue, different from conventional chondrosarcoma. It characteristically contains undifferentiated mesenchymal cells admixed with mature chondrocytes. Due to its low histological grade, survival is 89% at 10 years.^[19]^ Moreover, thyroidectomy does not have a significant impact on survival for thyroid metastases. If isolated metastasis to the thyroid or signs of tracheal compression, surgery should be suggested first.^[23]^ Although the prognosis is poor, surgery also helps diagnose the primary tumor and preserve the quality of life.

## 5 Conclusions

Overall, the thyroid gland is an infrequent site of metastasis from chondrosarcoma. In the view of this uncommon association, it may lead to the delay in establishing the correct diagnosis, especially when the primary site has been resected many years before and/or thyroid gland metastasis presents initially. Therefore, FNAB and immunohistochemistry are essential markers for thyroid tumors. Thus, whenever a neoplasm is assessed in the thyroid gland, and/or the patient has a history of previously diagnosed chondrosarcoma, careful clinic, ultrasound and radiological evaluation are recommended in spite of its relatively low occurrence. Additionally, the treatment of thyroid metastasis from chondrosarcoma depends on histological subtypes of chondrosarcoma and timing, number, location, and extension of metastasis.

## Author contributions

**Writing – original draft:** Zhi-Hong Wu, Jin-Yao Dai, Jia-ni Shi, Mei-Yu Fang, Jun Cao.

**Writing – review & editing:** Zhi-Hong Wu, Jin-Yao Dai, Jia-ni Shi, Mei-Yu Fang, Jun Cao.

## References

[1] Lam KY Lo CY Metastatic tumors of the thyroid gland: a study of 79 cases in Chinese patients. Arch Pathol Lab Med 1998;122:37–41. 9448014

[2] Haugen BR Nawaz S Cohn A Secondary malignancy of the thyroid gland: a case report and review of the literature. Thyroid 1994;4:297–300. 7833666 10.1089/thy.1994.4.297

[3] Sisu AM Tatu FR Stana LG Chondrosarcoma of the upper end of the femur. Rom J Morphol Embryol 2011;52:709–13. 21655665

[4] Simon F Classe M Vironneau P Thyroid compressive mass, a metastasis of femur chondrosarcoma after 14 years: case report and literature review. Braz J Otorhinolaryngol 2017;83:602–4. 26774495 10.1016/j.bjorl.2015.09.008 PMC9444784

[5] Ortiz S Tortosa F Sobrinho Simoes M An extraordinary case of mesenchymal chondrosarcoma metastasis in the thyroid. Endocr Pathol 2015;26:33–6. 25510635 10.1007/s12022-014-9351-6

[6] Darouassi Y Touati MM Chihani M Chondrosarcoma metastasis in the thyroid gland: a case report. J Med Case Rep 2014;8:157. 24886665 10.1186/1752-1947-8-157 PMC4038081

[7] El Otmany A Hafid H Hamada H Métastase intrathyroïdienne d’un chondrosarcome. Médecine Du Maghreb 2001;87:53–4.

[8] Bakx PA van den Ingh HF Baggen RG An unusual metastasis of a chondrosarcoma in the thyroid gland. Eur J Surg 1993;159:643–4. 8130309

[9] Ishikawa M Hirano S Tsuji T Management of metastasis to the thyroid gland. Auris Nasus Larynx 2011;38:426–30. 21239125 10.1016/j.anl.2010.11.009

[10] Wood K Vini L Harmer C Metastases to the thyroid gland: the Royal Marsden experience. Eur J Surg Oncol 2004;30:583–8. 15256229 10.1016/j.ejso.2004.03.012

[11] Willis RA Metastatic Tumours in the Thyreoid Gland. Am J Pathol 1931;7:187–208. 183. 19969962 PMC2062637

[12] Evola FR Costarella L Pavone V Biomarkers of Osteosarcoma, Chondrosarcoma, and Ewing Sarcoma. Front Pharmacol 2017;8:150. 28439237 10.3389/fphar.2017.00150 PMC5383728

[13] Wang Z Chen G Chen X Predictors of the survival of patients with chondrosarcoma of bone and metastatic disease at diagnosis. J Cancer 2019;10:2457–63. 31258751 10.7150/jca.30388 PMC6584356

[14] Rozeman LB Hogendoorn PC Bovee JV Diagnosis and prognosis of chondrosarcoma of bone. Expert Rev Mol Diagn 2002;2:461–72. 12271817 10.1586/14737159.2.5.461

[15] Ferguson JL Turner SP Bone cancer: diagnosis and treatment principles. Am Fam Physician 2018;98:205–13. 30215968

[16] van Maldegem AM Gelderblom H Palmerini E Outcome of advanced, unresectable conventional central chondrosarcoma. Cancer 2014;120:3159–64. 24995550 10.1002/cncr.28845

[17] Calzolari F Sartori PV Talarico C Surgical treatment of intrathyroid metastases: preliminary results of a multicentric study. Anticancer Res 2008;28 (5b):2885–8. 19031929

[18] Papi G Fadda G Corsello SM Metastases to the thyroid gland: prevalence, clinicopathological aspects and prognosis: a 10-year experience. Clin Endocrinol 2007;66:565–71. 10.1111/j.1365-2265.2007.02773.x 17371476

[19] Gelderblom H Hogendoorn PC Dijkstra SD The clinical approach towards chondrosarcoma. Oncologist 2008;13:320–9. 18378543 10.1634/theoncologist.2007-0237

[20] Brown RE Boyle JL Mesenchymal chondrosarcoma: molecular characterization by a proteomic approach, with morphogenic and therapeutic implications. Ann Clin Lab Sci 2003;33:131–41. 12817616

[21] Nakamura T Matsumine A Yamada S Oncological outcome after lung metastasis in patients presenting with localized chondrosarcoma at extremities: Tokai Musculoskeletal Oncology Consortium study. Onco Targets Ther 2016;9:4747–51. 27536136 10.2147/OTT.S107638 PMC4973757

[22] Evans HL Ayala AG Romsdahl MM Prognostic factors in chondrosarcoma of bone: a clinicopathologic analysis with emphasis on histologic grading. Cancer 1977;40:818–31. 890662 10.1002/1097-0142(197708)40:2<818::aid-cncr2820400234>3.0.co;2-b

[23] De Ridder M Sermeus AB Urbain D Metastases to the thyroid gland-a report of six cases. Eur J Intern Med 2003;14:377–9. 14769497 10.1016/s0953-6205(03)90005-7

